# Tracking migration and health inequities

**DOI:** 10.2471/BLT.23.290776

**Published:** 2023-12-14

**Authors:** Elisa Mosler Vidal, Kolitha Prabash Wickramage

**Affiliations:** a Centre on Migration, Policy and Society, University of Oxford, 58 Banbury Rd, Park Town, Oxford OX2 6QS, England.; b Migration Health Division, International Organization for Migration, Geneva, Switzerland.

Over 281 million people around the world are counted as international migrants.^1^ Many migrants are forcibly displaced – with 36.4 million refugees and 6.1 million asylum-seekers by mid-2023. Furthermore, there were 62.5 million internally displaced people at the end of 2022.^2^ While many of these migrants are healthy, many, in particular refugees, asylum-seekers and internally displaced people, are at risk of poor health outcomes and often experience health-related inequities, facing little or no access to health care.

Addressing this risk through inclusive health systems is vital to achieving universal health coverage (UHC) and is in line with existing rights conventions. Inclusive health systems can also have positive effects on integration. Moreover, while expanding health-care access is fundamental, policy-makers must simultaneously address the wider social determinants of migrants’ health. However, policy opportunities are constrained by a lack of timely and quality data. Generating and using more reliable data is necessary to advance migration health and achieve better public health outcomes for all.

## Uneven access and outcomes

While the aim of UHC applies to individuals irrespective of legal status, some countries do not offer health-care services to migrants. Considerable national variability exists regarding health-care access and health policies for migrants; some countries provide similar levels as they do for citizens and/or the native born, while others exclude migrants from life-saving care, primary health-care services, vaccination and/or health promotion interventions. Data covering 84 countries showed that in half of the countries, migrants were granted the same access to health services as nationals in official policies, while in 37% of the countries included in the study this was contingent on migrants’ legal status.^3^ Most bilateral labour agreements and memoranda of understanding on migration worldwide do not include provisions that cover health benefits for migrants; a study analysing 144 such agreements reported that only 30% of these did.^4^ Even if migrants are offered health-care services, discrimination or barriers that may be administrative or related to language or culture can lead to low uptake of services. Some migrants, such as refugees and asylum-seekers, may encounter particular issues, for example related to a lack of interpretation and cultural mediation services. 

Ensuring migrants have access to quality health care would improve their health status as well as integration prospects, for example, by strengthening their ability to engage in meaningful employment. Moreover, health system inclusivity has wider positive impacts; equal health-care access is linked to economic growth and can increase employment and productivity.^5^ Restricting refugee health-care access is expensive in the long-term, while extending care is linked to savings.^6^

However, health is shaped by more than just health-care access. Migration is recognized as a social determinant of health and can affect many factors that shape everyday life and well-being, such as education, employment, social protection and housing. Changing policy to address these factors, such as to improve migrant labour market integration, could also help improve health outcomes. Addressing key structural factors that influence migrants’ well-being is especially important in the context of the protracted nature of many conflicts and long duration of much displacement. Moreover, discussing and designing new policies on issues such as asylum-seekers’ right to work or foreign qualification recognition could have other positive impacts on countries’ health systems. For example, doing so could boost migrants’ fiscal contributions, which in many contexts already contribute significantly to health systems. In Switzerland, for example, the national social security system could collapse by 2027 if migration were halved.^7^ Furthermore, such policies could increase migrants’ skills contributions; in many countries, foreign-born medical personnel are crucial to the national health workforce.

## Patchy evidence and data gaps

Regular, quality data on migration and health are lacking, as migrants, including refugees, are largely invisible in official data relating to health. This gap makes it difficult to understand health inequities and any differences in health outcomes between migrants and others. Available evidence is patchy. For example, many migrant workers are in precarious contexts, yet their health status and health determinants remain poorly understood. In 2021, the International Labour Organization occupational safety and health statistics database disaggregated by migratory status data on only 13 out of 79 countries.^8^ In the absence of quality official statistics, applied research becomes critical to drive evidence-based policies and programmes. However, less than 1% (162/21 457) of research on international migration health published between 2000 and 2016 was from low-income countries.^9^ Overall, little regular data collection on migrants’ health status takes place; the same applies to data collection on migration-related determinants of health, how migrants navigate health care in practice, and other topics. Data on migrants in and from low- and middle-income countries are especially lacking.

Often, discourse around migration health outcomes, including from advocacy actors who state that all migrants are vulnerable, overlooks nuance. Migrants are a heterogenous group and major differences in health exist across subgroups. Nevertheless, detailed data on the health outcomes of migrants that reveal these differences, for example examining how factors related to sex, gender, employment and other areas interact with migratory status to affect health, is usually missing. Several reasons for this lack of data exist. Routine data collection often does not include migration variables. Furthermore, there is often little integration between existing data sources, low data comparability and poor representativeness of migrants in existing data. To change this situation, national authorities must collect, analyse and report data on health outcomes and burden of disease by key characteristics including migratory status, sex, age and disability. Household surveys can generate rich data on migration by introducing migration modules, and new approaches can link data sets and ensure stronger data protection.

Improving data is not always enough. With migration governance highly politicized and much discourse othering migrants, public opinion is a major determinant of migration policy today. Policy-makers can disregard evidence in seeking to appease constituencies. Therefore, creativity is essential when communicating information to engage with different audiences effectively. Practical examples of effective models of migrant health-care financing in other countries such as from Thailand, which uses a voluntary migrant-paid premium,^10^ could help motivate policy-makers.

## What can be done?

Countries can learn from existing good practices. Some countries that are long-term hosts of large populations of displaced people have taken steps to reduce health inequities for refugees. For example, Uganda hosts the largest number of refugees in Africa, with approximately 1.5 million, and is among the top refugee-hosting countries in the world. The country provides an example of extensive refugee integration throughout public health policy, programmes and infrastructure, improving health care for refugees and host communities alike. The Ugandan authorities provide refugees with access to education and extended the right to work and start a business, among others, to address various underlying social determinants of migration health.^11^ With help from international organizations, refugees were integrated into existing village and district health-care systems, which were strengthened to promote UHC. An evaluation showed high sustainability of health-care services beyond the regular life cycle of humanitarian assistance, and after agencies such as the United Nations High Commissioner for Refugees eventually handed over management of several services back to local authorities.^12^ Finally, the Ugandan government includes refugees in routine national data collection, including on health.^13^

## The way forward

The overarching goal of *Transforming our world: the 2030 agenda for sustainable development*, that is leaving no one behind, is unrealistic if migrant populations continue to be restricted in their abilities and rights to access health services. Governments need to address this situation; the number of people on the move will continue to grow due to the consequences of climate change and ongoing and new conflicts, among many other reasons. In September 2023, the Sustainable Development Goals Summit underlined how promises made to realize global goals, from tackling diseases to achieving UHC and improving migration data, remain unmet. Meeting these objectives requires countries to provide access for all migrants to safe, quality and affordable health care; establish migrant-sensitive programmes to address barriers to care; think holistically about migrants’ health to tackle its social determinants; and develop the capacity of health systems. Doing so also requires strengthening countries’ capacity to regularly collect, analyse and report quality data on migration health. Besides supporting inclusive policy, a stronger evidence base would open up possibilities for data and research to focus on other links between migration and health; for example, mobility trends of health workers and how diaspora populations may impact health in origin countries. 

Finally, to advance policy and bridge the gap between knowledge and action, dialogue on migration and health must be increasingly linked, with more collaboration between the migration and health policy spheres. Doing so would help mainstream migration into public health and vice versa, fostering transformational change. Similarly, stronger collaboration between actors and sectors within countries is crucial, for example through intersectoral working groups. Boosting cross-border collaboration is also key. International cooperation on migration is too often zero sum; this approach is of limited use when considering that promoting migration health is necessary to the health of all. Cooperation must consider migrants’ health; for example, more health-care provisions in bilateral labour agreements and memoranda of understanding could be included. Cooperation should include Skills Mobility Partnerships for medical professionals, agreements between countries that offer training and skills development, help meet labour needs, build health system capacity and over time, address health inequities between countries. Finally, improved collaboration on migration health can be framed as a collective action issue. The global community should ask itself why so little is done to improve migrants’ health, although this is understood to be vital to public health, and what can be done to change this situation. 
